# Hunger

**Published:** 1877-12

**Authors:** 


### HUNGER.

If a man in good health has not eaten anything for some days, he will die if he eats heartily. When persons are found in an almost starving condition, light food, in small quantities, and at short intervals, is essential to safety. The reason is, that as soon as we begin to feel hungry, the stomach rolls and works about, and continues to do so, unless satisfied, until it is so exhausted that there is scarcely any vital energy; it is literally *almost tired to death*, and, therefore, digestion is performed slowly, and with great difficulty. Hence, when a person has been kept from eating several hours beyond his usual time, instead of eating fast and heartily, he should take his food with deliberation, and only half as much as if he had eaten at the regular time. Sudden and severe illness has often resulted from the want of this precaution, and sometimes death has followed.

